# Differential Response to Local Stimulator of Interferon Genes Agonist Administration in Tumors with Various Stimulator of Interferon Genes Statuses

**DOI:** 10.3390/cancers17020175

**Published:** 2025-01-08

**Authors:** Alina Drzyzga, Justyna Czapla, Sybilla Matuszczak, Barbara Łasut-Szyszka, Tomasz Cichoń, Ewelina Pilny, Magdalena Jarosz-Biej, Ryszard Smolarczyk

**Affiliations:** Center for Translational Research and Molecular Biology of Cancer, Maria Skłodowska-Curie National Research Institute of Oncology, 44-102 Gliwice, Poland; justyna.czapla@gliwice.nio.gov.pl (J.C.); sybilla.matuszczak@gliwice.nio.gov.pl (S.M.); barbara.lasut-szyszka@gliwice.nio.gov.pl (B.Ł.-S.); tomasz.cichon@gliwice.nio.gov.pl (T.C.); ewelina.pilny@gliwice.nio.gov.pl (E.P.); magdalena.jarosz-biej@gliwice.nio.gov.pl (M.J.-B.)

**Keywords:** stimulator of interferon genes (STING), cancer immunotherapy, STING agonist, poorly immunogenic tumors, inflammation

## Abstract

**Background/Objectives**: The stimulator of interferon genes (STING) is currently accepted as a relevant target for anti-cancer therapies. Besides encouraging results showing STING agonist-induced tumor growth inhibition, in some types of tumors the effect is less prominent. We hypothesized that higher STING levels in cancer cells and the possibility of its activation determine a greater anti-cancer response. As the local administration of STING agonists induces a systemic reaction, we emphasized the importance of host tumor-induced hematological disruption in the efficiency of the therapeutic response. **Methods**: We investigated the response to STING stimulation in murine cancer cell lines—melanoma (B16-F10) and breast carcinoma (4T1)—and murine normal cell lines: fibroblast cells (NIH/3T3), endothelial cells (H5V), and macrophages (J774A.1). We assessed STING agonist-induced tumor growth inhibition and the therapy’s impact on the hematological system parameters and systemic cytokine release. **Results**: Our results underlined the improved therapeutic effect of STING activation in melanoma (B16-F10) over breast carcinoma (4T1) tumors. The outcomes reflected a high dysregulation of the hematological system in mice with developed 4T1 tumors, which may support persistent inflammation and impede STING-induced therapeutic effects. Moreover, among typical cytokines produced following STING activation, CCL2 fold change was the one that increased the most in the serum of B16-F10-bearing mice and differentiated the observed response to the STING agonist between investigated tumor models. **Conclusions**: The current study provides new evidence of the different responses to STING activation among two poorly immunogenic tumor models. The high abundance of STING in B16-F10 cells and the possibility of its activation is linked with improved therapeutic response in vivo compared to 4T1. The effect also seems to be connected with a less dysregulated hematological system in mice with B16-F10 tumors over mice with 4T1 tumors. This highlighted the need for general insight into tumor-induced local and systemic responses to the efficiency of the proposed therapy.

## Simple Summary

The activation of the stimulator of interferon genes (STING) is one way to boost the immune system’s fight against cancer. However, clinical trials assessing the anti-cancer potential of STING agonists encounter limitations because of STING protein status in tumor tissue and the inability to evoke a sufficient immune response. In this study, we aimed to assess the impact of STING level and the possibility of its activation in two murine cancer cell lines, melanoma (B16-F10) and breast carcinoma (4T1), on the anti-cancer effect of STING-targeting therapy. We showed that a STING agonist induced the improved tumor growth inhibition of B16-F10 cells over 4T1 tumors; enhanced STING expression and activation in B16-F10 cells; intensified hematologic dysfunction in 4T1-bearing mice; and increased the systemic release of CCL2 in B16-F10 over 4T1-bearing mice. This study shows the importance of deep insights into STING level and the expected effects of its activation in the context of a disturbed hematological system.

## 1. Introduction

Nowadays, immunotherapy, along with surgery, radiotherapy, chemotherapy, and precision therapy, constitutes an equal pillar of oncological strategies. Among immune-stimulating therapies, targeting the cyclic GMP-AMP synthase (cGAS)—stimulator of interferon genes (STING) pathway has emerged as an encouraging anti-cancer approach. cGAS-STING is activated when dsDNA occurs in the cell cytosol. As a result, 2′3′-GMP–AMP cyclic dinucleotide (2′3′-cGAMP, cGAMP) is produced, which triggers the STING protein and enables its translocation to the Golgi apparatus. There, STING recruits the TANK-binding kinase 1 (TBK1) which enables the phosphorylation of STING and IFN regulatory factor 3 (IRF3), leading to the expression of type I interferons. Additionally, STING activation leads to the activation of nuclear factor kappa B transcription factor (NF-kB), which induces the expression of cytokines such as IL-6, TNFα, and T cell-attracting chemokines [1,2,3].

In multiple mouse tumor models, the activation of STING leads to the activation of innate immune cells and provides an anti-tumor response. Thus, the activation of the cGAS-STING pathway has become the subject of numerous studies in the field of cancer therapy [4,5,6]. This boosts the design and synthesis of novel compounds targeting the STING pathway, with some having reached the clinical trial level. However, clinical trials examining the potential of STING-targeting compounds have reported limited or even zero anti-cancer response [4,7] https://clinicaltrials.gov (accessed on 28 July 2024). Similarly, in pre-clinical models of aggressive and refractory tumors, STING agonists have limited potential and require combination with additional therapeutic agents to achieve the inhibition of tumor growth [8,9]. Additionally, there has been an observed decrease in STING expression in multiple tumor tissues and even reductions in its level during cancer progression. This correlates with a poor prognosis [10,11,12,13]. Therefore, the full understanding of the variables and events necessary for sufficient anti-cancer response is of great significance.

Importantly, cancer is considered a systemic disease. Its progress is linked with complex changes in the immune and hematologic systems [14]. The disruption of hematopoiesis emerges primarily in the expansion of immature and immunosuppressive neutrophils and monocytes. In cancer patients, a systemic increase in the frequencies of regulatory T and B cells and a decrease in CD8^+^, CD4^+^ T, and dendritic cells are described. Moreover, the cytotoxic potential of innate immune cells declines [15].

The aim of this study was to assess if the level of STING and its activation in cancer cells determines the magnitude of the anti-cancer therapeutic response, which could be observed at the level of systemic changes in cytokine and hematological parameters. As a part of our research, we studied the different responses to STING stimulation in two cancer cell lines and the corresponding murine tumor models: melanoma (B16-F10) and breast carcinoma (4T1). Local treatment with 2′3′-cGAMP induces a systemic immune response; thus, besides tumor morphological changes and growth inhibition, we have also assessed the early changes in parameters of complete blood count and serum cytokines.

In this pre-clinical study, we confirmed that the level of STING, as well as the possibility of its activation in cancer cells, determines the anti-cancer therapeutic response. Importantly, the study emphasizes the systemic response to local STING stimulation which varies depending on the degree of the hematologic and immune system disruption. The current research provides new insight into different responses to STING agonists between two poorly immunogenic tumor models.

## 2. Materials and Methods

### 2.1. Cell Lines and Therapeutic Agent

Murine melanoma B16-F10 and breast cancer 4T1 cell lines (ATCC, Manassas, VA, USA) were cultured in RPMI 1640 (Biowest, Nuaillé, France) supplemented with 10% heat-inactivated fetal bovine serum (EURx, Gdańsk, Poland) and 1% penicillin–streptomycin (Biowest). Murine fibroblast NIH/3T3, endothelial cells H5V, and macrophages J774A.1 were cultured in DMEM high glucose (Biowest), supplemented as above. Cell cultures were maintained in standard conditions (37 °C, 5% CO_2_, 95% humidity) and passaged with 0.25% trypsin-EDTA (Biowest). Cultures were routinely tested for the presence of Mycoplasma.

2′3′-cGAMP VacciGrade™ (cyclic guanosine monophosphate–adenosine monophosphate, cGAMP, InvivoGen, Toulouse, France) was used as a therapeutic agent.

### 2.2. Cell Viability

Cells were placed in 96-well plates and incubated for 48 h. The cells were treated with 2′3′-cGAMP at a concentration of: 0.1; 1; 10; 100 µM in the corresponding medium and cultivated for 24 h. Cell viability was analyzed using MTS assay (Promega, Madison, WI, USA) according to the manufacturer’s instructions.

### 2.3. Western Blot Analysis

Cells were treated with 10 µM 2′3′-cGAMP in the corresponding medium for 24 h. The cells were lysed with the IP buffer (Thermo Fisher Scientific, Waltham, MA, USA) supplemented with protease and phosphatase inhibitors (Merck, Darmstadt, Germany). The lysates were separated by SDS-PAGE and electro-transferred onto PVDF membranes. The membranes were blocked with 5% nonfat dry milk and incubated with primary antibodies: anti-STING (clone: D2P2F, Cell Signaling Technology, Danvers, MA, USA), anti-phospho-STING (Ser365) (clone: D8F4W, Cell Signaling Technology) and anti-HSC70 (clone: B-6, Santa Cruz Biotechnology, Dallas, TX, USA). All incubations with primary antibodies were performed overnight at 4 °C. HRP-conjugated polyclonal secondary antibodies (Thermo Fisher Scientific) were detected by chemiluminescence (Thermo Fisher Scientific).

### 2.4. Conditioned Medium Assessment

Cells were washed three times with PBS- and treated with 10µM 2′3′-cGAMP in a medium without the addition of fetal bovine serum. Cells were cultivated for 24 h (NIH/3T3) or 48 h (J774A.1, H5V, 4T1, B16-F10). The conditioned medium was collected and centrifuged at 10,000× *g*, 5 min. The type and quantity of cytokines have been assessed with LEGENDplex™ Mouse Inflammation Panel (BioLegend, San Diego, CA, USA) by BD FACSCanto II flow cytometer (Becton Dickinson (BD), Franklin Lakes, NJ, USA), according to the manufacturer’s instructions. The data were analyzed using LEGENDplex™ Data Analysis Software Version 8.0 (BioLegend).

### 2.5. Mice and Ethics Statement

Experiments were conducted on female mice BALB/c and C57Bl/6NCrl (8 to 10 weeks old), obtained from Charles River Breeding Laboratories (Wilmington, MA, USA). Experiments on animals were carried out according to the recommendations in the Guide for the Care and Use of Laboratory Animals of the National Institutes of Health and 3R rules, with the consent of the Local Ethics Commission of Animal Experiments in Katowice (permission No. 49/2021 and 26/2024). The mice were housed in the Maria Skłodowska-Curie National Research Institute of Oncology, Gliwice Branch (Poland) in a pathogen-free facility (SPF standard). The mice received a total pathogen-free standard and complete diet (Altromin International, Lage, Germany).

### 2.6. Murine Tumor Model and Therapy

Murine B16-F10 and 4T1 cells were injected subcutaneously (lower flank) with 2 × 10^5^ cancer cells. The tumors were measured with calipers and tumor volumes were determined using the formula: volume = width^2^ × length × 0.52. 2′3′-cGAMP VacciGrade™ was injected intratumorally at a dose of 5 µg/mice in 100 μL of PBS. The mice in the control group received 100 μL of PBS.

### 2.7. Tumor Collection and Histochemical Staining

On the 14th day after cancer cell inoculation, the mice were terminated and tumors were collected into OCT (Leica Biosystems, Wetzlar, Germany), frozen in liquid nitrogen, and stored at −80 °C. The frozen sections (5 µm) were examined histochemically (hematoxylin/eosin staining, Merck, Darmstadt, Germany), and the analysis was conducted using the Nikon Eclipse 80i microscope (Nikon Instruments Inc., Tokyo, Japan).

### 2.8. Complete Blood Count Assessment

Mice blood withdrawal from the submandibular vein was conducted 12 h after intratumoral 2′3′-cGAMP injection. The blood was collected in EDTA-coated tubes. Parameters were assessed with hematology analyzer scil Vet abc Plus+ (Mouse Research settings) (scil Vet abc Plus+, Horiba, Warsaw, Poland).

### 2.9. Serum Cytokines Assessment

The blood was drawn from deeply anesthetized mice 12 h after intratumoral 2′3′-cGAMP injection by cardiac puncture. The serum was separated and the type and quantity of cytokines were assessed using the LEGENDplex™ MU Cytokine Release Syndrome Panel (BioLegend) by BD FACSCanto II flow cytometer, according to the manufacturer’s instructions. The data were analyzed using LEGENDplex™ Data Analysis Software Version 8.0 (BioLegend). The level of IFN-β was examined using LEGEND MAX™ Mouse IFN-β ELISA Kit (BioLegend) according to the manufacturer’s instructions.

### 2.10. Statistical Analysis

The results were statistically analyzed using Statistica software version 12 (TIBCO Software Inc., StatSoft Polska, Kraków, Poland). The normality of the distribution was verified with the Shapiro–Wilk test. The homogeneity of variance was checked using the Brown–Forsythe and/or Levene’s tests. Student’s *t*-test or the Mann–Whitney U-test was used to compare two groups of variables. Kruskal–Wallis test with Dunn’s multiple comparison post hoc test or one-way analysis of variance with appropriate post hoc was used to compare more than two groups of variables. Statistical significance of parametric and non-parametric tests was marked with asterisks and hashtags, accordingly. *p*-values < 0.05 were considered statistically significant. The data are shown as mean ± SEM (Standard Error of Mean) or mean ± SD (Standard Deviation).

## 3. Results

### 3.1. Macrophages and Endothelial Cell Lines Constitute Major cGAMP-Responders In Vitro and B16-F10 Cells Show Greater Responsiveness on STING Agonist than 4T1 Cells

**Figure 1:**
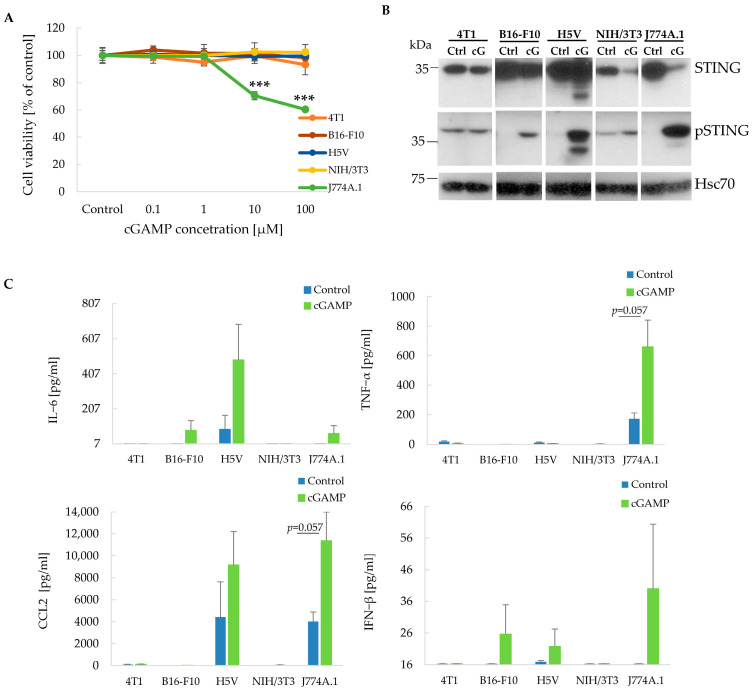
The impact of 2′3′-cGAMP on cell viability, STING level and activation, and cytokine production. (**A**) MTS assay of cell viability. Cells were treated with different 2’3’-cGAMP concentrations. *** *p* < 0.001 vs. Control, 0.1 µM and 1 µM groups, ANOVA with post hoc HSD Tukey’s. The data from 3 independent experiments are shown, mean ± SD (Standard Deviation), *n* = 4. (**B**) Western Blot analysis of STING and phospho-STING levels in cells treated with 10 µM 2’3’-cGAMP. Hsc70 was used as a loading control. (**C**) Level of selected cytokines produced by cells treated with 10 µM 2’3’-cGAMP. Analyzes were conducted using the LEGENDplex™ Mouse Inflammation Panel with a flow cytometer. The minimum values on the *y*-axis are the lower measured limit. The maximum values on the *y*-axis for CCL2 are the upper measured limit. The data are shown as mean + SEM (Standard Error of Mean), and the data were assessed using the Mann–Whitney U-test.

The anti-tumor efficacy of the STING agonist depends on multiple factors. Among them, the ability of cancer cells to respond to STING agonists plays an essential role. Considering this issue, we assessed the impact of STING agonists on murine 4T1 breast carcinoma, B16-F10 melanoma, H5V endothelial, NIH/3T3 fibroblasts, and J774A.1 macrophage cell lines. Only in the case of macrophages did 2′3′-cGAMP have an impact on the cell viability. STING agonist-induced 30% and 40% cell viability decrease at a concentration of 10 and 100 µM, appropriately (Figure 1A). Western blot analysis revealed that the tested cell lines were characterized by different levels of STING (Ctrl). 4T1 and NIH/3T3 cells showed lower STING levels compared to the other tested cell lines. In B16-F10, NIH/3T3, and J774A.1 cells, a reduction in STING level was observed after incubation with 2′3′-cGAMP relative to the control lysates (STING lane). 2′3′-cGAMP induced STING activation (pSTING lane) in B16-F10, J774A.1, H5V and NIH/3T3 cells. The strongest STING activation was observed in J774A.1 and H5V cells (Figure 1B). The uncropped blots are shown in Supplementary Figure S1. The current analysis showed STING agonist-induced production of the following cytokines: IL-6, CCL2, TNF-α, and IFN-β. 2′3′-cGAMP increased level of IL-6 in B16-F10 (10.7 times vs. control), H5V (5.5 times vs. control) and J774A.1 (8.5 times vs. control) cells, TNF-α in J774A.1 (3.8 times vs. control) cells, CCL2 in H5V (2 times vs. control) and J774A.1 (2.8 times vs. control) cells, and IFN-β in B16-F10 (1.6 times vs. control), H5V (1.3 times vs. control) and J774A.1 (2.5 times vs. control) cells (Figure 1C).

### 3.2. STING Agonist Inhibits the Growth of B16-F10 Melanoma More Effectively than 4T1 Breast Carcinoma

**Figure 2:**
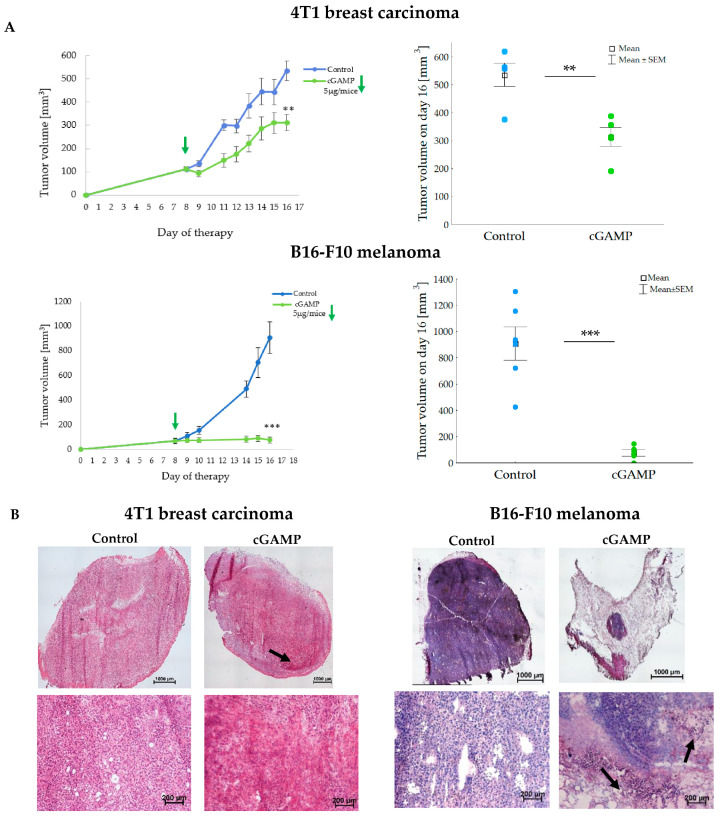
The impact of 2’3’-cGAMP on the growth and morphological changes in B16-F10 and 4T1 tumors. (**A**) Single dose of 5 µg/mice 2’3’-cGAMP was intratumorally administered. The data are shown as mean ± SEM (left panel). Graph showing tumor volume on the last day of the experiment. The data are shown as raw data and mean ± SEM (right panel), ** *p* < 0.01; *** *p* < 0.001 Student’s *t*-test. (**B**) Representative photographs of H&E staining of tumor 12 h post-treatment. Whole slide images (upper panel) were combined from 12 to 20 single photographs (5× magnification). Representative photographs of tumors at higher magnification (10× lens magnification) are shown on the lower panel. The area of hemorrhagic necrosis (4T1 breast carcinoma) and the influx of inflammatory cells (B16-F10 melanoma) are indicated by black arrows.

The difference between 4T1 and B16-F10 cancer cells’ response to the STING agonist led us to the verification of the results in vivo. STING agonists inhibited the growth of both types of tumors. The mean volume of 2’3’-cGAMP-treated 4T1 tumors was 1.7 times smaller than the control tumors (~300 vs. 535 mm^3^). 2’3’-cGAMP was more effective in the growth inhibition of B16-F10 tumors. The mean volume of B16-F10 tumors treated with 2’3’-cGAMP was 11.9 times smaller than in the control group (~77 vs. 910 mm^3^) (Figure 2A). As STING agonist had been shown to have a rapid impact on tumor growth inhibition [16], the morphological changes in the tumors were assessed 12 h after treatment. In 4T1 tumors there were no significant structural changes after treatment with 2’3’-cGAMP (Figure 2B, left panel). In the periphery of the tumor, small areas of hemorrhagic necrosis (black arrow) were observed. In B16-F10 tumors, 2’3’-cGAMP induced extensive structural changes (Figure 2B, right panel). The tumor tissue was distorted, with the areas of infiltrating immune cells (black arrows).

### 3.3. The Presence of 4T1 Tumor Increases Granulopoiesis While STING Agonist Decreases the Platelet Parameters and the Total Count of White Blood Cells

**Figure 3:**
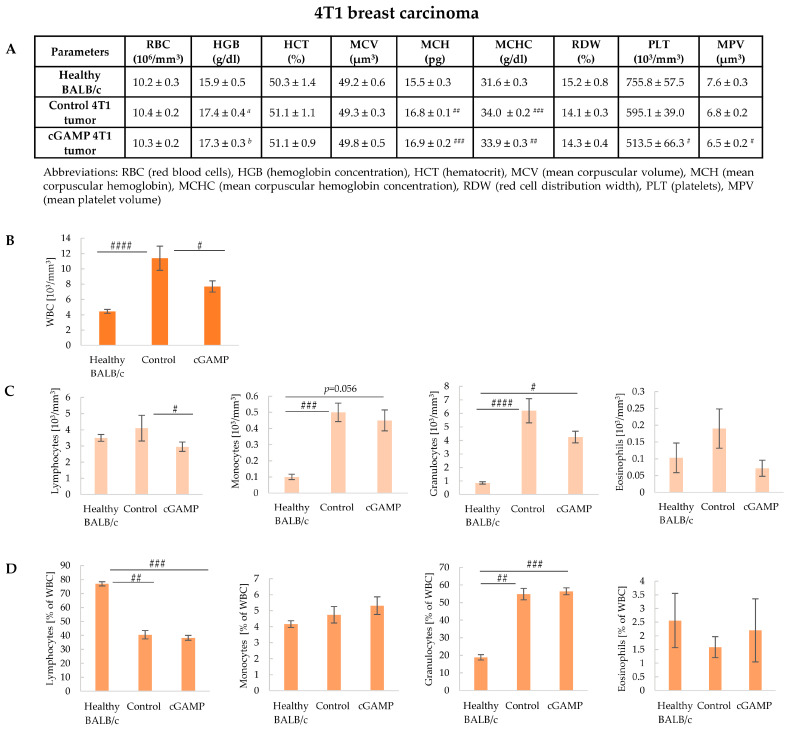
Parameters of complete blood count in BALB/c healthy and with 4T1 control and 2’3’-cGAMP-treated-tumor mice. The analyses of mice were conducted 12 h after 2’3’-cGAMP administration. The data from two independent experiments are shown as mean ± SEM, *n* = 5. (**A**) Parameters of red blood cells and platelets. The statistical significance compared to healthy BALB/c mice # *p* < 0.05; ## *p* < 0.01; ### *p* < 0.001; *a*: *p* = 0.059; *b*: *p* = 0.062 Kruskal–Wallis test with Dunn’s multiple comparison post hoc test. (**B**) The total count of WBC # *p* < 0.05; #### *p* < 0.0001 Kruskal–Wallis test with Dunn’s multiple comparison post hoc test. (**C**) The total number of individual WBC populations, # *p* < 0.05; ### *p* < 0.001; #### *p* < 0.0001 Kruskal–Wallis test with Dunn’s multiple comparison post hoc test. (**D**) The percentage of individual WBC populations, ## *p* < 0.01; ### *p* < 0.001 Kruskal–Wallis test with Dunn’s multiple comparison post hoc test.

To verify the impact of the local STING agonist administration on the systemic response the parameters of complete blood count have been analyzed and compared to the results of healthy mice. The attendance of the 4T1 tumor increased the parameters of red blood cells, the total count of WBC (white blood cells), and granulocytes (Figure 3). Mice with 4T1 tumors, control, and 2’3’-cGAMP-treated demonstrated higher results of hemoglobin and its parameters (MCH and MCHC) compared to the healthy mice. 2’3’-cGAMP administration led to a decrease in the platelet count and its parameter (MPV) in comparison to the healthy mice (Figure 3A). We observed an increased number and percentage share of granulocytes in both control and 2’3’-cGAMP-treated mice compared to the healthy mice (Figure 3C,D). However, due to increased granulopoiesis during 4T1 tumor development, there was a decrease in the lymphocyte percentage in mice with control and 2’3’-cGAMP-treated tumors in comparison to the healthy mice (Figure 3D), although no changes in the number of lymphocytes in mice with 4T1 control tumors in comparison to the healthy mice was noted (Figure 3C,D). Moreover, the administration of 2’3’-cGAMP led to a systemic decrease in the number of lymphocytes in comparison to mice with control tumors. We reported an increase in the number of blood monocytes in mice with 4T1 tumors compared to the results of healthy mice, even though monocyte percentage share showed no difference. There was no difference revealed in the number and percentage share of eosinophils between groups (Figure 3C,D).

### 3.4. STING Agonist Administration Decreases the Number of Blood Lymphocytes with a Simultaneous Increase in the Blood Granulocytes and Eosinophils Share in Mice with B16-F10 Tumors

**Figure 4:**
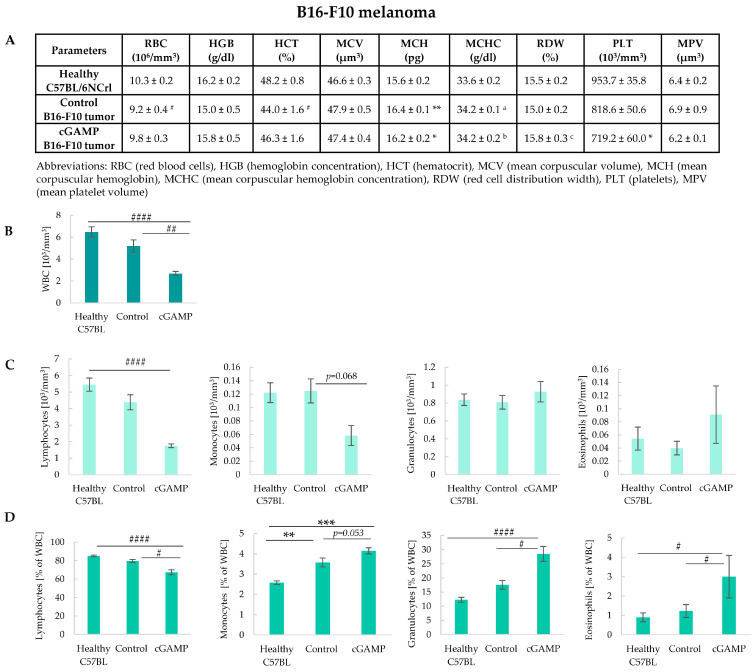
Parameters of complete blood count in C57BL/6NCrl healthy and with B16-F10 control and 2’3’-cGAMP-treated mice with tumors. The analyses of mice were conducted 12 h post 2’3’-cGAMP administration. The data from two independent experiments are shown as mean ± SEM, *n* = 5. (**A**) Parameters of red blood cells and platelets. The statistical significance compared to healthy mice: # *p* < 0.05 Kruskal–Wallis test with Dunn’s multiple comparison post hoc test; * *p* < 0.05, ** *p* < 0.01 Tukey’s HSD test; a: *p* < 0.05 Scheffé test; b: *p* < 0.05 LSD test; The statistical significance compared to Control B16-F10 tumor: c: *p* < 0.05 Tukey’s HSD test. (**B**) The total number of WBC ## *p* < 0.01; #### *p* < 0.0001 Kruskal–Wallis test with Dunn’s multiple comparison post hoc test. (**C**) The total number of individual WBC populations, ####* p* < 0.0001 Kruskal–Wallis test with Dunn’s multiple comparison post hoc test. (**D**) The percentages of individual WBC populations, # *p* < 0.05; #### *p* < 0.0001 Kruskal–Wallis test with Dunn’s multiple comparison post hoc test; ** *p* < 0.01;*** *p* < 0.001 Tukey’s HSD test.

Breast carcinoma 4T1 and melanoma B16-F10 tumors differ in, among others, their strain of origin, tumor microenvironment, and cancer cell-intrinsic STING presence and response to STING agonists. The presence of B16-F10 tumor decreased RBC count and the value of HCT in comparison to healthy mice. Mice with both control and 2’3’-cGAMP-treated tumors have increased hemoglobin parameters (MCH and MCHC) compared to healthy mice. 2’3’-cGAMP administration led to a decrease in the number of platelets in comparison to healthy mice (Figure 4A). In mice treated with 2’3’-cGAMP, 2.4 times decrease in the total WBC count compared to healthy animals and a 1.9 times decrease compared to mice with control tumors were noted (Figure 4B). 2’3’-cGAMP treatment led to a systemic decrease in lymphocytes compared to healthy and control tumor-bearing mice (Figure 4C,D). Mice with tumors (both control and 2’3’-cGAMP-treated) were shown to increase monocyte percentage compared to healthy mice (Figure 4D). However, its number in the blood of 2’3’-cGAMP-treated mice decreased (Figure 4C). In the case of granulocytes, even though their total number did not change (Figure 4C) due to the 2’3’-cGAMP-induced decrease in the number of lymphocytes (mostly contributing to total WBC), we reported an increase in granulocyte percentage in mice with 2’3’-cGAMP-treated tumors compared to other groups (Figure 4D). Similar results were observed in the case of eosinophils, which constituted the minor population of cells among WBC (Figure 4C,D).

### 3.5. Intratumoral STING Agonist Administration Induce Systemic Release of Multiple Cytokines in 4T1 Tumor-Bearing Mice

**Figure 5:**
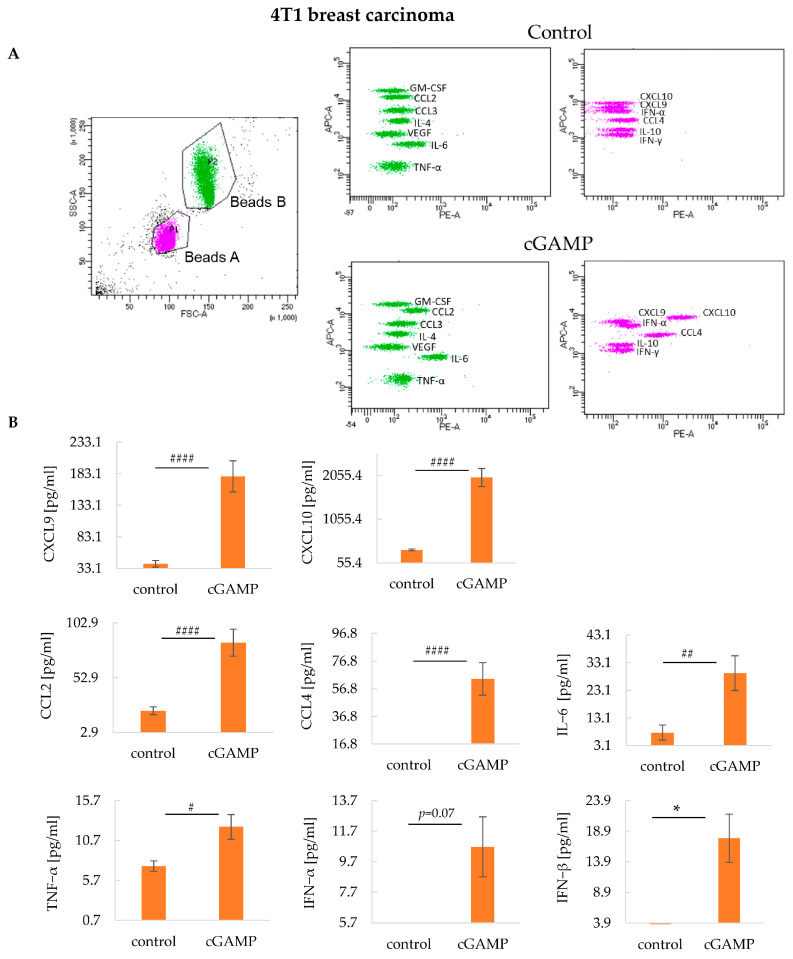
The impact of intratumoral 2’3’-cGAMP administration on the systemic release of cytokines in 4T1 tumors. Cytokines have been analyzed in serum collected 12 h post 2’3’-cGAMP administration. Analyzes were conducted using the LEGENDplex™ MU Cytokine Release Syndrome Panel. (**A**) Representative graphs showing flow cytometry gating and signals from corresponding cytokines. (**B**) The minimum values on the *y*-axis are the lower measured limit for the individual cytokines. Graphs of cytokines with statistically irrelevant data are not shown. IFN-β concentration was examined using LEGEND MAX™ Mouse IFN-β ELISA Kit. The data from the two independent experiments are shown as mean ± SEM, *n* = 3; # *p* < 0.05; ## *p* < 0.01; #### *p* < 0.0001 Mann–Whitney U-test; * *p* < 0.05 Student’s *t*-test.

It is well established that the anti-tumor effect of STING agonists depends on the induction of multiple cytokines. We determined if local intratumoral administration of 2’3’-cGAMP affects inflammatory cytokine release in the systemic circulation. Among tested cytokines, the most 2’3’-cGAMP-induced increase in chemokines secretion was observed in CXCL9 and CXCL10. 2’3’-cGAMP induced 4.4 times (vs. control) and 5.7 times (vs. control) increase in CXCL9 and CXCL10, respectively. We also reported an increase in CCL2 (3.7 times vs. control), CCL4 (3.8 times vs. control), IL-6 (3.7 times vs. control), and TNF-α (1.7 times vs. control) after 2’3’-cGAMP treatment. Moreover, a slight increase in type I interferons (IFN-α and IFN-β) in STING agonist-treated mice was noted (Figure 5A,B) (Table S1).

### 3.6. Intratumoral STING Agonist Administration Induces More Extensive Systemic Release of CCL2 in Mice with B16-F10 Tumors than in Mice with 4T1 Tumors

**Figure 6:**
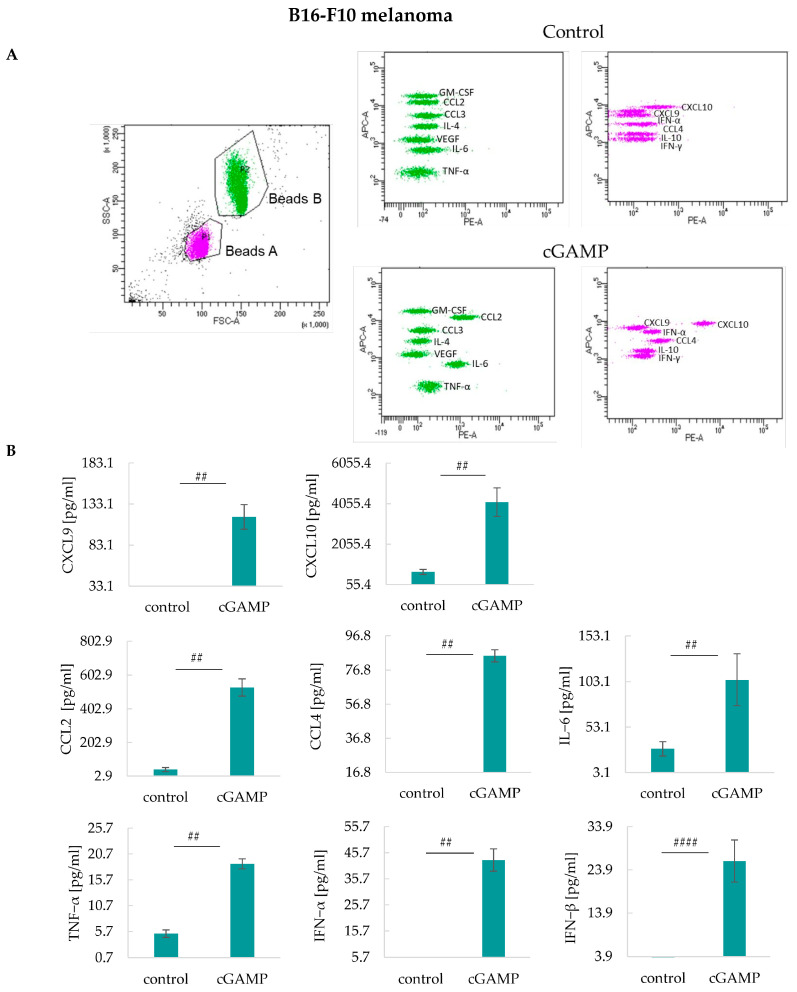
The impact of intratumoral 2’3’-cGAMP administration on the systemic release of cytokines in B16-F10 tumors. Cytokines have been analyzed in serum collected 12 h post 2’3’-cGAMP administration. Analyzes were conducted using the LEGENDplex™ MU Cytokine Release Syndrome Panel. (**A**) Representative graphs showing flow cytometry gating and signals from corresponding cytokines. (**B**) The minimum values on the *y*-axis are the lower measured limit for the individual cytokines. Graphs of cytokines with statistically irrelevant data are not shown. IFN-β concentration was examined using LEGEND MAX™ Mouse IFN-β ELISA Kit. The data from one independent experiment are shown as mean ± SEM, *n* = 3. Each experiment was repeated; ## *p* < 0.01; #### *p* < 0.0001 Mann–Whitney U-test.

Likewise, in 4T1 tumor-bearing mice, substantial production of various cytokines after 2’3’-cGAMP treatment was noted in B16-F10 tumor-bearing mice (Figure 6B). We reported a significant, higher than in 4T1 tumor-bearing mice, 2’3’-cGAMP-induced increase in CXCL10, CCL2, CCL4, TNF-α, IFN-α and IFN-β concentrations. 2’3’-cGAMP induced 3.5 times (vs. control) and 6 times (vs. control) increase in CXCL9 and CXCL10 levels, respectively. 2’3’-cGAMP treatment led to an increase in the production of CCL2 (12.7 times vs. control), CCL4 (5.1 times vs. control), IL-6 (3.6 times vs. control), and TNF-α (3.6 times vs. control). Moreover, in STING agonist-treated mice, an increase in type I interferons concentration (IFN-α and IFN-β) was observed (Figure 6A,B) (Table S1).

## 4. Discussion

In recent years, therapies targeting the cGAS-STING pathway have gained scientists’ interests. The pathway activation results in the production of multiple pro-inflammatory cytokines, which boost strong immune response and, in some cases, provide tumor growth inhibition. However, although benefits have been observed in preclinical tests, the translation of STING agonists into clinics encountered limitations [4,17]. In the current study, we have assessed the impact of STING activation on two poorly immunogenic “cold” tumor models. First, we focused on the cancer cells’ STING expression and the possibility of its activation. Next, we underlined the impact of STING agonists on tumor growth inhibition and systemic response taking into account tumor-induced hematologic system disruption.

A number of reports indicate the importance of STING expression in the prediction of clinical outcomes. The magnitude of the response to STING activation depends on its expression and, due to the post-translational modifications, on the possibility of its activation [11,12,13,18]. In the current research, J774A.1 macrophages were the only cell line showing reduced viability after STING stimulation. It is well known that STING agonists lead to the immune cells death. cGAS-STING pathway activation induces apoptosis of T lymphocytes [19,20,21], malignant and normal B cells [22], and activates lysosomal cell death of monocytes [23] and macrophages [24]. Moreover, STING activation switches the macrophages toward the M1-cytotoxic phenotype. However, besides their anti-cancer functions, such macrophages are more susceptible to STING-mediated necroptosis [25,26]. In some cancers, STING expression and its post-translational modifications are proposed to be a predictive indicator of patients’ survival and therapy response. Lohinai et al. showed an increased overall survival (OS) of patients with non-small cell lung cancer (NSCLC) classified as “STING-positive”. Moreover, STING expression was shown to be lost with increasing tumor stage [12]. In another study, STING methylation was correlated with unfavorable outcomes [13]. Biesaga et al. also pointed to an improvement in the disease-free survival rate of patients with high STING expression within the oral cavity and oropharyngeal squamous cell carcinoma tumors [11]. In our study, among cancer cell lines, 4T1 cells were characterized by a low level of STING expression. Simultaneously, the highest level of STING was observed in B16-F10 melanoma, H5V endothelial cells, and J774A.1 macrophages. The highest cGAMP-induced STING phosphorylation was noticed in normal cells: macrophages and endothelial cells. Among TME cells, macrophages have been shown to express higher levels of INFβ following STING activation than dendritic, endothelial, and T cells [16]. On the other hand, Demaria et al. indicated tumor endothelial cells as primary contributors to STING-mediated anti-tumor response [27]. Similarly, Yang et al. revealed an increase in CD8^+^ T lymphocyte infiltration and improved OS of patients with augmented STING expression in tumor vasculature [28]. Among tested cancer cell lines melanoma cells showed enhanced STING phosphorylation compared to breast carcinoma cells. Interestingly, in cGAMP-responsive cells (B16-F10, H5V, NIH/3T3, J774A.1) we observed a reduction in the STING expression. This phenomenon might have resulted from STING degradation, which prevented excessive cytokines production and uncontrolled immune response [29,30]. Additionally, unlike in B16-F10 cells, in 4T1 cells similar level of STING phosphorylation in both control and cGAMP-treated cells was observed. That may be related to sustained weak inflammation which supports tumor growth and may boost therapy resistance. B16-F10, unlike 4T1 cells, exhibits a subtle release of the cytokines typical for a STING pathway. Interestingly, the strongest cGAMP-responding cells—H5V endothelial cells and J774A.1 macrophages—secrete different types of cytokines. Among other cells present in TME, dendritic cells are considered essential in STING-dependent anti-tumor response [31]. Additionally, cGAMP treatment has been shown to activate NK cells and unleash their killing capacity [32,33]. Thus, apart from assessing the presence of STING in tumor and stromal cells, it also seems crucial to know the composition of TME.

In the current study, both tumor models are considered poorly immunogenic, characterized by immune anergy and resistance to immunotherapy [34,35,36]. Nevertheless, both types of tumors responded to cGAMP treatment, with B16-F10 tumors being more susceptible to STING activation. That has been observed via tumor growth inhibition and extensive structural damage. On the contrary, cGAMP-treated 4T1 tumors, besides regions of hemorrhagic necrosis, have no structural changes. In other studies conducted on mice tumor models, STING agonist-induced hemorrhagic necrosis and inhibition of tumor growth were observed [37,38]. Based on the current results, the different responses on STING activation may partly result from the STING level in cancer cells, a different mice strain, and hence various genetic background [39].

The efficacy of currently proposed anti-cancer immunotherapies depends on proper communication between TME and circulating immune cells. Importantly, most immune cells are short-lived cells that are continuously replenished from appropriate progenitor cells. Importantly, a developing tumor leads to hematopoiesis abnormalities, which contribute to immunosuppression [40]. Investigating STING-activating therapy on the systemic response, we observed the different impacts of tumor presence and its targeting on the parameters of blood components between analyzed tumor models. The presence of the 4T1 tumor increased hemoglobin levels and the total number of WBC, and induced a shift in the major percentage-share from lymphocytes observed in healthy individuals, toward granulocytes observed in tumor-bearing mice. These results indicate an intensified process of granulopoiesis during 4T1 tumor development. Thus, therapies aiming to rebalance this proportion may bring advantages. Interestingly, in the case of STING activation, in 4T1 tumor-bearing mice, a decrease in the number of circulating granulocytes, lymphocytes, monocytes, and platelets was observed. Considering the second tumor model, we should note that the different genetic backgrounds of B16-F10 (C57BL/6NCrl strain) and 4T1 (BALB/c strain) tumors have a remarkable impact on the immune system and hematological dysregulation. Thus, we observed distinct effects of STING agonists on blood count parameters between these tumors. Similar to our results, other authors also observed the influence of melanoma tumors on the occurrence of anemia and dysregulation of the immune system [41]. In melanoma-bearing mice, the use of STING agonist led to thrombocytopenia and reduction in the total WBC count, which was related to the drop in the number of lymphocytes and monocytes These data indicate that the local administration of the STING agonist led to the systemic change at the cellular level, which is mediated through complex cytokine—cell communication. STING-activating therapies are also known to convert immunologically “cold” tumors into “hot” ones. In melanoma tumors, we and others observed STING agonist-induced infiltration of NK and CD8^+^ T cells and macrophage polarization toward the M1 anti-tumor phenotype. These events are most evident in the later phase of the immune response and provide tumor growth inhibition [5,8,27,33,42,43,44]. Unlike in 4T1 tumor-bearing mice, the use of STING agonists in B16-F10 melanoma increases the share of granulocytes and eosinophils. This is associated with the cGAMP-induced shift within the percentage share of individual WBC populations. Namely, a cGAMP-induced decrease in lymphocyte count caused an increased percentage share of the other WBC populations.

In this study, we observed different serum cytokine profiles between mice with melanoma and breast carcinoma tumors. Namely, mice with melanoma tumors demonstrated higher levels of CXCL10, IL-6, and CCL2 in the serum compared to mice with control breast carcinoma tumors. Otherwise, in the case of TNFα and CXCL9, mice with control breast carcinoma tumors exhibited increased levels of these cytokines than melanoma-bearing mice. Both assessed here, tumor types differ in mouse strain, genetic background, and TME [39]. As we showed, the level of STING differs between studied tumors and cancer cells, which may impact the immune cells and cytokines profiles in the tumor milieu and systemic circulation [8,39]. However, in both tumor types, STING agonist-induced serum cytokines changed at a similar level. The pivotal effect of STING activation is the production of cytokines. Among them, type I interferons and CXC motif chemokines are known to be crucial in the anti-cancer immune response. Here, we showed that local STING agonist administration induces substantial cytokine production in systemic circulation. STING activation primarily induced the production of CXCL9 and CXCL10. Their role as T lymphocyte-attracting proteins is widely known [45,46]. We and the others observed increased T-cell infiltration into the tumor following STING activation [5,8,16,46,47,48]. However, while the increase in T lymphocyte-attracting chemokines was observed in the serum of both tumor models analyzed here, STING-dependent T cell infiltration is not always noticed. It might be masked by the STING-induced overall CD8^+^ T cells decrease or might be dependent on the tumor milieu [8,49]. Besides the increased production of anti-tumor cytokines: TNF-α, IFN-α, and IFN-β, STING activation is linked with elevated levels of IL-6. IL-6 in multiple research have been shown to exhibit a pleiotropic effect. Besides their supporting impact on cancer cell proliferation, survival, and metastatic dissemination, some reports indicate IL-6-mediated activation and proliferation of T lymphocytes. Sharma et al. found in a murine model of melanoma, the necessity of IL-6 for effective priming of CD8^+^ T lymphocytes in the tumor-draining lymph nodes [50,51]. Therefore, increased IL-6 production may reduce the therapeutic effect of STING activation or may support immune activation. The recent discoveries showed that the blockage of IL-6 signaling enhances the anti-tumor effect of STING-targeting therapies [52,53,54]. Additionally, we observed an increase in CCL2 and CCL4 concentration following STING activation. Among analyzed cytokines, CCL2 fold change was the one that increased the most remarkably (12.7 times vs. control) in the melanoma model in comparison to the breast carcinoma model (3.7 times vs. control). During inflammation, CCL2 controls the increase and activation of monocytes and macrophages [55]. Further, depending on the circumstances, it may induce macrophage polarization toward M1 or M2 phenotype [56,57,58]. Nevertheless, in tumors, CCL2 was shown to induce an increase in tumor-supporting TAMs (tumor-associated macrophages) and MDSCs (myeloid-derived suppressor cells). Moreover, a CCL2 blockade has been shown to shift TAMs toward anti-tumor M1-like phenotype [3,59,60]. Thus, it seems possible that the production of CCL2 may increase macrophage infiltration and simultaneously interrupt their M1-polarization, diminishing the STING therapeutic effect. Wan et al. have shown that the combination of STING activation with CCR2 blockage exerts anti-tumor immunity in pancreatic cancer [3]. Similar to our study, Perera et al. observed that STING agonists stimulate cytokines production in plasma and peripheral tissues following intratumoral administration [61]. Strong induction of chemoattractive cytokines following STING stimulation may justify the benefits of the combination of STING-activating agents with adoptive cell therapy [62,63]. Interestingly, Wu et al. showed a STING-dependent mechanism of tumor-immune evasion in adoptive T-cell transfer. Thus, multiple variables need to be considered when planning such therapeutic strategies [64].

A significant limitation in the use of STING agonists, especially naturally occurring cGAMP, is their short duration, due to degradation by enzymes such as ENPP1 [65]. Therefore, most STING agonists must be administered locally to the tumor, which limits their use in hard-to-reach tumors and allows application to narrow types of tumors. Many of the first-generation agonists, currently undergoing clinical trials, are injected intratumorally (e.g., NCT04592484; NCT04020185; NCT04167137). Currently, many studies are being conducted in search of more stable STING agonists [65]. One of the most promising compounds is MSA-2. It is a non-nucleotide STING agonist, which can be administered orally [66,67]. MSA-2 is not an active ligand but after noncovalent dimerization becomes a pharmacologically active ligand, that acts as naturally occurring cGAMP and thus can be suitable for systemic administration in the clinic.

## 5. Conclusions

Currently, it is well proven that STING agonists induce strong inflammation and act as vaccine adjuvants. The potent response on STING activation fostered researcher focus on this pathway and led to overcoming some obstacles like the potential of too excessive cytokine release [43,68]. Based on the current research, we state that the magnitude of STING agonists’ anti-tumor activity partly depends on the cancer cells’ intrinsic STING expression. The high level of STING in B16-F10 cells and the possibility of its activation are linked with observed in vivo improved therapeutic response compared to 4T1. The development of tumors affects hematological profiles in various ways. Unlike B16-F10 melanoma, 4T1 breast carcinoma development strongly influences mice hematological parameters, with intensified granulopoiesis being the most prominent sign. Here, STING activation provides a stronger anti-tumor response in B16-F10 tumors than in 4T1 tumors. In 4T1 tumors, STING targeting does not overcome sustained weak inflammation which supports breast carcinoma growth. More and more data suggest that the patient’s hematological system condition is extremely important for the effectiveness of the therapy. That pushes the attention to the broad insight into the patient’s state, which may improve the development of personalized therapies. Even though this approach requires consideration of numerous variables, it seems rational and, due to inter alia AI development, also feasible.

## Data Availability

The data that support the findings of this study are available from the corresponding author, R.S. and A.D., upon reasonable request.

## Supplementary Materials

The following supporting information can be downloaded at: https://www.mdpi.com/article/10.3390/cancers17020175/s1, Figure S1: Uncropped blots of STING protein after cGAMP stimulation; Table S1: Cytokine amounts after cGAMP stimulation
