# Supplementary material for: Differential Response to Local Stimulator of Interferon Genes Agonist Administration in Tumors with Various Stimulator of Interferon Genes Statuses
Source: Cancers (Basel). 2025 Jan 8;17(2):175. doi: 10.3390/cancers17020175 (PMC11763682; doi:10.3390/cancers17020175)
Supplement: Supplementary file 1 [file cancers-17-00175-s001.zip › cancers-3330665-supplementary.docx]

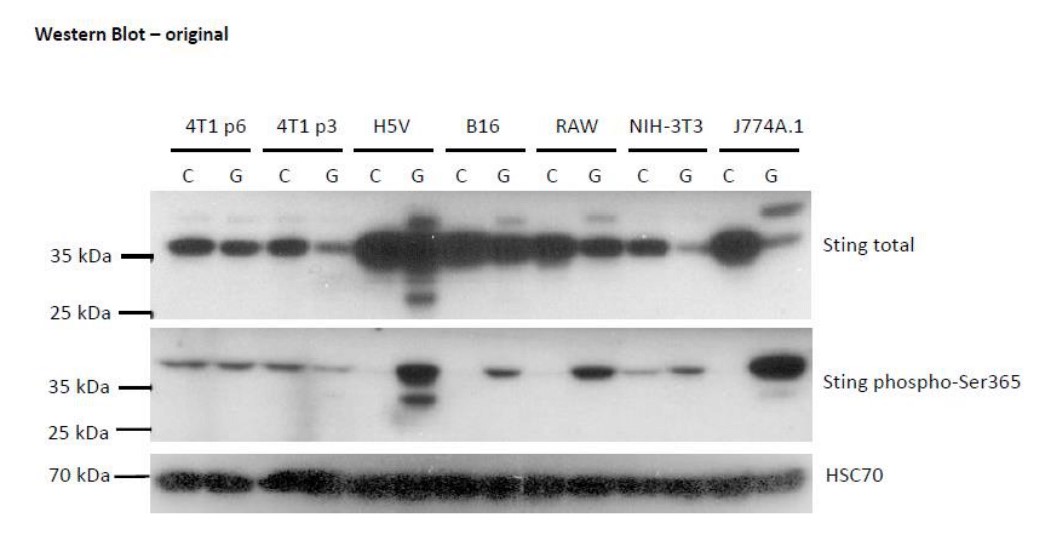


Figure S1. Uncropped blots of STING protein after cGAMP stimulation.

| 4T1 |  |  | |  |  |  |  |  |  |  |  |  |  |  |
| --- | --- | --- | --- | --- | --- | --- | --- | --- | --- | --- | --- | --- | --- | --- |
|  | IFN-γ | | IL-10 | CCL4 (MIP-1β) | IFN-α | CXCL9 (MIG) | CXCL10 (IP-10) | TNF-α | IL-6 | VEGF | IL-4 | CCL3 (MIP-1α) | CCL2 (MCP-1) | GM-CSF |
| Control | 4,66 | 182,96 | | 16,78 | 5,69 | 40,41 | 353,01 | 7,48 | 7,79 | 2,11 | 1,02 | 12,63 | 22,71 | 15,34 |
| cGAMP | 4,66 | 182,96 | | 63,96 | 10,67 | 178,83 | 2009,24 | 12,41 | 29,32 | 2,11 | 1,02 | 12,63 | 84,86 | 15,34 |
| SE Control | 0 | 0 | | 0 | 0 | 5,32 | 21,03 | 0,65 | 2,76 | 0 | 0 | 0 | 3,64 | 0 |
| SE cGAMP | 0 | 0 | | 11,77 | 1,97 | 24,56 | 205,55 | 1,54 | 6,21 | 0,03 | 0,00 | 0,83 | 12,34 | 0 |
| Fold change | 1 | 1 | | 3,81 | 1,88 | 4,43 | 5,69 | 1,66 | 3,77 | 1 | 1 | 1 | 3,74 | 1 |

| B16-F10 |  |  |  |  |  |  |  |  |  |  |  |  |  |
| --- | --- | --- | --- | --- | --- | --- | --- | --- | --- | --- | --- | --- | --- |
|  | IFN-γ | IL-10 | CCL4 (MIP-1β) | IFN-α | CXCL9 (MIG) | CXCL10 (IP-10) | TNF-α | IL-6 | VEGF | IL-4 | CCL3 (MIP-1α) | CCL2 (MCP-1) | GM-CSF |
| Control | 4,66 | 182,96 | 16,78 | 5,69 | 33,07 | 684,70 | 5,26 | 29,27 | 2,11 | 1,02 | 12,63 | 41,82 | 15,34 |
| cGAMP | 5,36 | 183,56 | 85,12 | 42,90 | 117,37 | 4125 | 18,80 | 105,05 | 2,11 | 1,02 | 12,63 | 529,54 | 15,34 |
| SE Control | 0,27 | 0 | 0 | 0 | 0 | 125,13 | 0,72 | 7,90 | 0 | 0 | 0 | 11,97 | 0 |
| SE cGAMP | 0,45 | 0,60 | 3,53 | 4,29 | 14,96 | 700,75 | 0,97 | 28,39 | 0 | 0 | 0 | 51,17 | 0 |
| Fold change | 1,15 | 1 | 5,07 | 7,54 | 3,55 | 6,02 | 3,57 | 3,59 | 1 | 1 | 1 | 12,66 | 1 |

Table S1. Cytokine amounts after cGAMP stimulation.
